# Smoking in Movies: A New Centers for Disease Control and Prevention Core Surveillance Indicator

**DOI:** 10.5888/pcd9.120261

**Published:** 2012-11-08

**Authors:** Tim McAfee, Michael Tynan

**Affiliations:** Author Affiliations: Michael Tynan, Centers for Disease Control and Prevention, Atlanta, Georgia.

Youth who are heavily exposed to onscreen smoking are approximately 2 to 3 times as likely to begin smoking as youth who are lightly exposed (1), and the Surgeon General concluded that there is a causal relationship between depictions of smoking in the movies and smoking initiation among young people (2). Among the 3 major motion picture companies with policies aimed at reducing tobacco-use incidents in their movies, the number of onscreen incidents per youth-rated movie (rated G, PG, or PG-13 by the Motion Picture Association of America) decreased 95.8% from 2005 through 2010 (3). These results appeared to indicate that movie companies were making progress at reducing smoking depictions in youth-oriented movies and that a company-by-company approach of adopting voluntary policies could be effective in nearly eliminating youth exposure to tobacco imagery in movies. However, new data from 2011 published by Glantz and colleagues (4) in *Preventing Chronic Disease* raise serious concerns about this individual company approach.

Glantz and colleagues found that in 2011, depictions of tobacco use per youth-rated movie rebounded; estimated instances of tobacco use in 2011 were more than one-third higher than in 2010 (4). Furthermore, the authors found that the largest increase in tobacco-use incidents in youth-rated movies was among the 3 movie companies that had produced the dramatic decline from 1995 through 2010 and had policies designed to discourage depictions of smoking in their movies. As a result of this sharp rebound, the difference in tobacco-use incidents per youth-rated movie between companies with policies and companies without policies diminished in 2011 (4). This difference suggests that individual company policies may not be sufficient to sustain a reduction in youth exposure to tobacco-use and other pro-tobacco imagery in movies and that more formal, industry-wide policies are needed.

The World Health Organization and other public health groups have recommended formal policies aimed at eliminating smoking in the movies (5,6). These policies include awarding an R rating to any movie with smoking or other tobacco-use imagery (with exceptions for portrayal of actual historical figures who smoked or the portrayal of negative effects of tobacco use), certifying that no payments have been received by studios for depicting tobacco use in movies, and ending the onscreen depiction of real tobacco brands.

Reducing smoking and tobacco use in youth-oriented movies is not a niche issue. The Surgeon General has concluded that there is a causal relationship between depictions of smoking in movies and smoking initiation among young people, and the US Department of Health and Human Services has set a goal of reducing youth exposure to onscreen smoking (7). Furthermore, among the nationwide goals set by Healthy People 2020, one of the objectives is the reduction of onscreen tobacco use imagery in youth-oriented movies and on television (8). These goals and objectives were set because the population-attributable risk associated with onscreen tobacco imagery is significant (9,10).

To assess progress toward the Healthy People 2020 objective, the Office on Smoking and Health at the Centers for Disease Control and Prevention (CDC) will now track and report annually on tobacco use imagery in youth-oriented movies as a core surveillance indicator by using the methods described in previous CDC publications (3,4). These data will be added to regular CDC reports to the public on smoking prevalence among youth and adults, total and per-capita cigarette consumption, and progress on tobacco control policies.

One of the major conclusions in the Surgeon General’s 2012 report on preventing tobacco use (2) was that after years of steady progress, declines in tobacco use by youth and young adults have slowed for cigarette smoking and have stalled for smokeless tobacco use (2). Each day in the United States, approximately 3,800 young people younger than 18 years smoke their first cigarette, and approximately 1,000 youth younger than 18 years become daily cigarette smokers (2). More than one-third of these smokers will eventually suffer and die from smoking-related illness. We all have a responsibility to prevent youth from becoming tobacco users, and the movie industry has a responsibility to protect our youth from exposure to tobacco use and other pro-tobacco imagery in movies that are produced and rated as appropriate for children and adolescents. Eliminating tobacco imagery in movies is an important step that should be easy to take.
